# Supplementary material for: Neuropeptide-dependent spike time precision and plasticity in circadian output neurons
Source: bioRxiv. 2024 Dec 21:2024.10.06.616871. Originally published 2024 Oct 7. Preprint. [Version 2] doi: 10.1101/2024.10.06.616871 (PMC11476009; doi:10.1101/2024.10.06.616871)
Supplement: Supplement 1 [file NIHPP2024.10.06.616871v2-supplement-1.pdf]

bioRxiv preprint doi: https://doi.org/10.1101/2024.10.06.616871; this version posted December 21, 2024. The copyright holder for this preprint (which was not certified by peer review) is the author/funder, who has granted bioRxiv a license to display the preprint in perpetuity. It is made available under aCC-BY 4.0 International license.

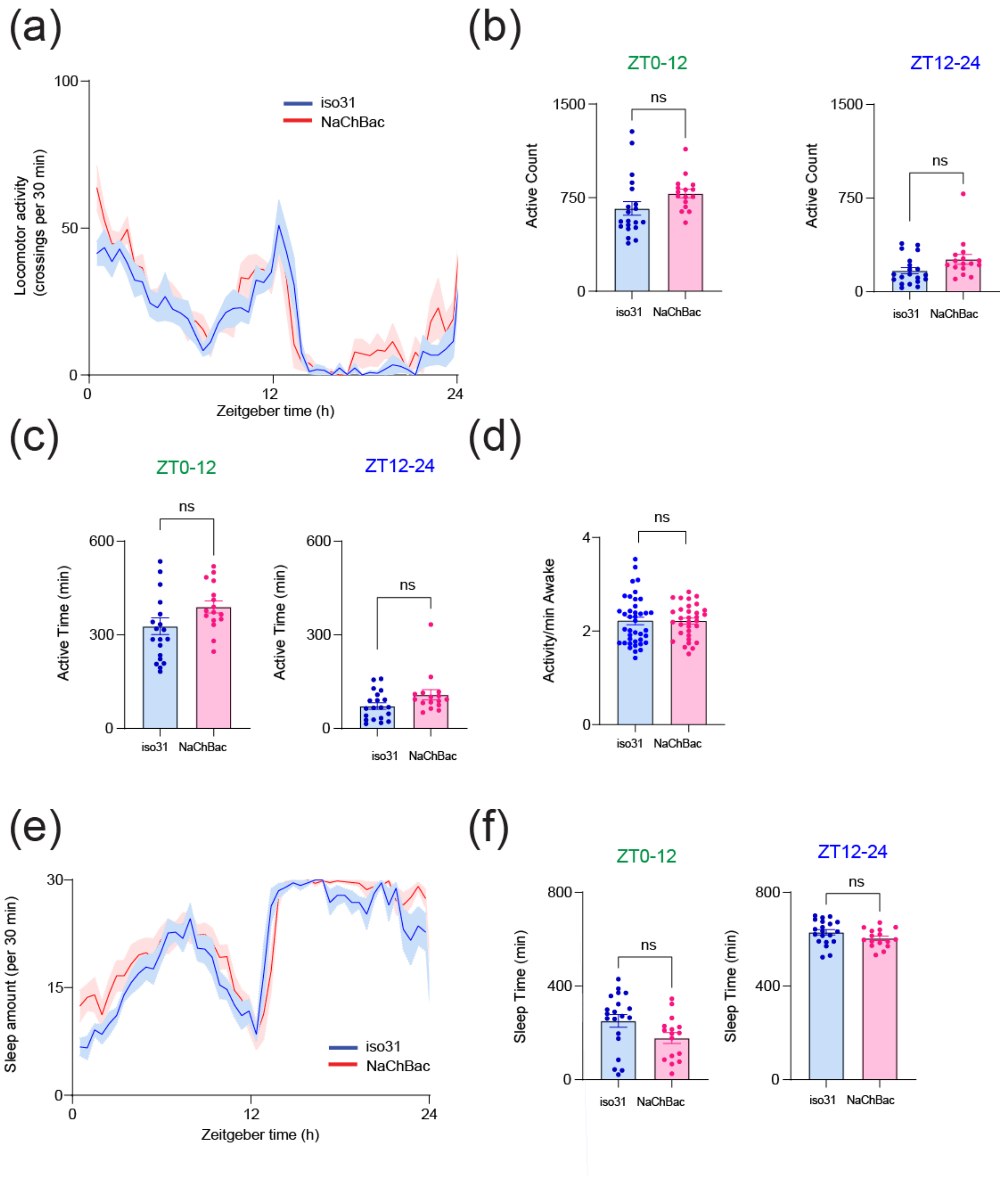

Supplementary Figure S1

Figure S1 (**a**) Activity profiles (**b**) Activity count at ZT0-12 and ZT12-24 **(c)** Active time at ZT0-12 and ZT12-24 (**d**) Daily waking activity of *iso31* (blue) and *UAS-NaChBac* alone control (red) flies. **(e)** Sleep profiles **(f)** Sleep time at ZT0-12 and ZT12-24 of *iso31* (blue) and *UAS-NaChBac*

bioRxiv preprint doi: https://doi.org/10.1101/2024.10.06.616871; this version posted December 21, 2024. The copyright holder for this preprint (which was not certified by peer review) is the author/funder, who has granted bioRxiv a license to display the preprint in perpetuity. It is made available under aCC-BY 4.0 International license.

alone control (red) flies. Sleep time plotted in 30 min bins. The statistics used were unpaired t-tests, and ns indicated non-significant.

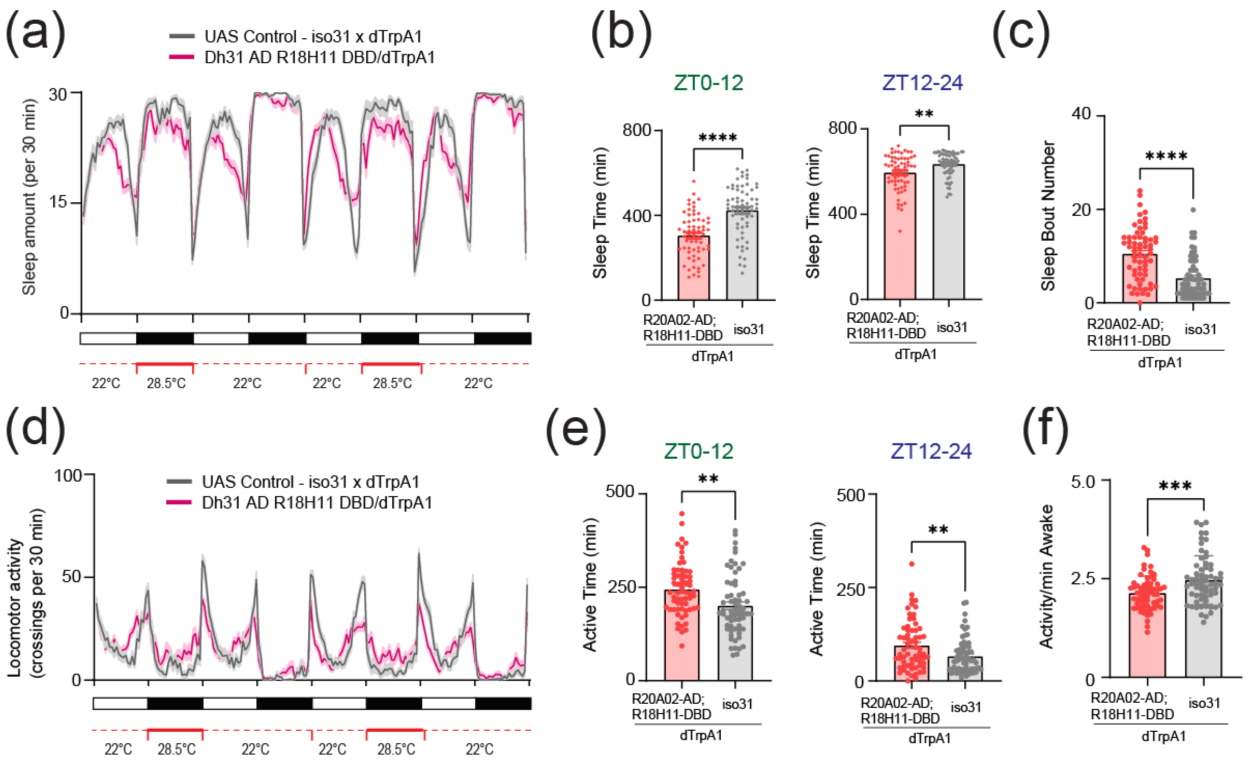

Supplementary Figure S2

Figure S2 (**a**) Sleep profiles (**b**) Sleep time at ZT0-12 and ZT12-24 (**c**) Sleep bout number of *UAS-dTRPA1>iso31* control (gray) and *R20A02-AD;R18H11-DBD>UAS-dTRPA1* (red) flies. (**d**) Activity profiles (**e**) Active time at ZT0-12 and ZT12-24 (**f**) Daily waking activity of *UAS-dTRPA1>iso31* control (gray) and *R20A02-AD;R18H11-DBD>UAS-dTRPA1* (red) flies. The statistics used were unpaired t-test with $^{**}p < 0.01$, $^{***}p < 0.001$ and $^{****}p < 0.0001$.
